# Optimized extraction of *Pinus taeda* knotwood sawmill residues as source of antifungal compounds

**DOI:** 10.1186/s40643-026-01072-x

**Published:** 2026-06-08

**Authors:** Rodrigo Coniglio, Juan Martín Rodao, Raquel Alonso, Facundo Fioritto, Karina Nicole Ruiz, Lucía Xavier, Jörn Appelt, María Noel Cabrera, Leonardo Clavijo

**Affiliations:** 1 Chemical Engineering Institute, Engineering School, Universidad de la República, Montevideo, Uruguay; 2 Mycology Laboratory, Sciences School, Universidad de la República, Montevideo, Uruguay; 3 Federal Research Institute for Rural Areas, Forestry and Fisheries, Institute of Wood Research, Johann Heinrich von Thünen-Institute, Hamburg, Germany

**Keywords:** *Pinus taeda*, Knotwood, Phenolic compounds, Antioxidant activity, Antifungal, Circular bioeconomy, Wood preservation

## Abstract

In Uruguay, *Pinus taeda* is the dominant conifer in industrial plantations, generating large volumes of knot-containing offcuts during processing. These residues are highly enriched in extractives, offering opportunities for valorization within a circular bioeconomy. This study examined the recovery of bioactive compounds from *P. taeda* knotwood through ethanol extraction, process optimization, Gas Chromatography coupled with Mass Spectrometry and Flame Ionization Detection (GC–MS/FID) analysis, and antifungal evaluation. The effects of extraction temperature, ethanol concentration, and liquid–solid (L/S) ratio on extraction yield, total phenolic content, and FRAP antioxidant activity were assessed, and response models were developed to identify optimal conditions. Maximum extraction yield (20.8%) occurred at 47 °C, 100% ethanol concentration, and L/S of 8.3. The highest phenolic content (5.7 g gallic acid equivalents/100 g) was predicted at 61 °C, 100% ethanol, and L/S 15, while the greatest antioxidant capacity (15.8 mmol ascorbic acid equivalents/100 g) was achieved at 75 °C and L/S 15, independently of ethanol concentration. Two extraction conditions, representing the best compromise among yield, phenolics, and antioxidant performance, were selected for further characterization and antifungal tests. GC–MS/FID analysis showed that stilbenes and terpenoids dominated the extracts. Antifungal assays against *Trametes versicolor* and *Gloeophyllum trabeum* revealed strong inhibition, reaching up to 65% and 97% after seven days. Overall, the results demonstrate that *P. taeda* knotwood residues can be efficiently valorised via ethanolic extraction to obtain bioactive fractions with high antioxidant and antifungal activity, supporting sustainable and circular approaches for wood protection.

**Graphical abstract:**

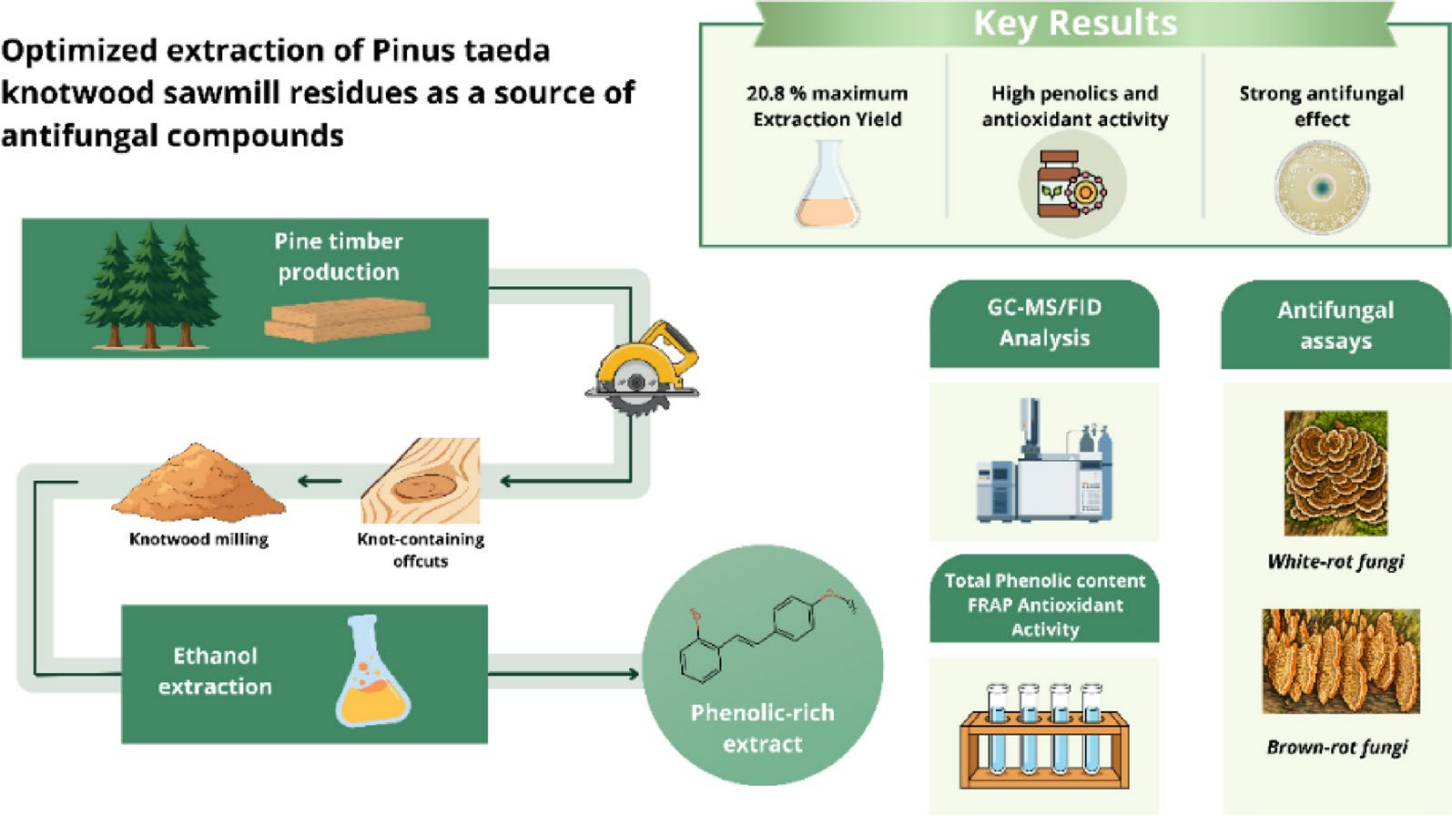

## Introduction

Wood has been used for centuries as a versatile construction and manufacturing material, and in recent decades its relevance has increased further, driven by the global search for renewable and environmentally friendly resources. In Uruguay, forest plantations expanded from 32,000 hectares in 1988 to 1.16 million hectares in 2024, of which about 75% are Eucalyptus species and 14% are pines, of which *Pinus taeda* (loblolly pine) dominates (Boragno 2025). Native to the southern United States and part of the “Southern yellow pine” group, it is the most widely planted pine in temperate regions worldwide because of its rapid growth and favourable properties, and it is used in a wide range of applications including light construction, beams, plywood, laminated timber, flooring and exterior uses (Dieste et al. 2018).

Knots, formed as woody tissue accumulates around branches, are embedded in the stem and appear in boards when logs are sawn (Parham and Gray 1984; Hillis 1987; Zink-Sharp 2003). They are the most important defect in sawn timber, reducing mechanical strength, altering appearance, and sometimes detaching during drying to form knotholes (Zink-Sharp 2003; Shmulsky and Jones 2011). Their presence also causes cracks and voids due to different drying behaviour, and knot size and distribution are among the first criteria for classification (Sandberg et al. 2023). In fact, knots remain the most common dominant defect in sawn timber, accounting for nearly 44% of all dominant defects detected in recent image-based analyses (Berezjuk et al. 2025). Furthermore, when coated with water-based paint, knots often cause yellow–brown discolorations known as knot bleeding, which result from the migration of highly concentrated extractives through the polymeric structure of the coating (Coniglio et al. 2023a, b). Therefore, knot sections constitute a distinctive by-product of sawmilling because of their high frequency and impact on board quality (Moore and Cown 2015).

Although problematic for timber production, knots are rich in extractives, often containing 20–30% and sometimes up to 40% of these secondary metabolites (Kebbi-Benkeder et al. 2015; Belt et al. 2017). Extractives are non-structural compounds with several functions and include phenolic compounds (stilbenes, lignans, flavonoids, tannins), terpenes and resin acids (Hillis 1987; Sjöström 1993; Nisula 2018). These molecules contribute directly to the natural durability of wood by acting as radical scavengers, metal chelators, enzyme inhibitors and antimicrobial agents (Valette et al. 2017; Santos et al. 2022; Kirker et al. 2024; Richard et al. 2024). Stilbenes such as pinosylvin are well documented for antifungal and antibacterial activity (Felhofer et al. 2018; Tran et al. 2023; Periferakis et al. 2025), lignans are highly concentrated in knots and exhibit antioxidant and antimicrobial properties (Willför et al. 2003a; Nisula 2018), flavonoids such as pinocembrin and taxifolin have strong radical scavenging activity (Rasul et al. 2013; Zheng et al. 2022; Intharuksa et al. 2024), and tannins inhibit fungal enzymes essential for lignin degradation. Several recent studies have confirmed these properties. For example, ethanolic extracts of *Pinus pinaster* knots, rich in lignans, stilbenes and flavonoids, strongly inhibited *Plasmopara viticola*, the causal agent of grapevine downy mildew, with pinosylvin derivatives and pinocembrin identified as the most active compounds (Gabaston et al. 2017). Extracts of *Pinus sylvestris* and *Pinus nigra* knotwood effectively inhibited both white-rot (*Trametes versicolor*,* Schizophyllum commune*) and brown-rot fungi (*Gloeophyllum trabeum*,* Fibroporia vaillantii*), with pinosylvin and its monomethyl ether confirmed as the main bioactive compounds (Vek et al. 2020). More recently, (Gérardin et al. 2023) reported that knot and branch wood extracts from silver fir, spruce and Douglas fir contain high amounts of polyphenols, particularly lignans and flavonoids, and display strong antioxidant, antibacterial and antifungal activities.

The low natural durability of *P. taeda* has traditionally been compensated in Uruguay by deep impregnation with chromated copper arsenate (CCA), which extends service life to 20 years (Dieste 2014), but this preservative is now banned in many countries due to health and environmental concerns (Chen and Olsen 2016; Morais et al. 2021). Other chemical preservatives such as pentachlorophenol, naphthalene or copper oxinate face similar restrictions (Miranji et al. 2022). This has intensified the search for safer and sustainable alternatives, including extracts from durable woods (Tascioglu et al. 2013) and pyrolysis distillates (Barbero-López et al. 2019), though using naturally durable woods is not sustainable at scale. In contrast, valorising underutilized residues such as knotwood offers a circular solution.

Wood-decay fungi are broadly classified into white-rot and brown-rot fungi. White-rot fungi degrade lignin as well as polysaccharides, leaving the wood fibrous and bleached (Daniel 2016) while brown-rot fungi primarily attack carbohydrates, causing rapid strength loss and leaving the wood brown and brittle (Belt et al. 2022; Qi et al. 2023). Both are relevant as model organisms in durability assessments. Knot extracts and their major compounds, especially pinosylvins, have shown inhibitory activity against both groups (Vek et al. 2020), highlighting the potential of knot residues as natural wood preservatives.

In this context, the aim of the present work is to explore the valorisation of *P. taeda* knotwood residues from sawmilling, within a circular economy framework. Specifically, the study aimed to (i) evaluate the influence of extraction temperature, ethanol concentration and liquid-solid (L/S) ratio on extraction yield (EY), total phenolic content (TPC) and FRAP antioxidant activity (FRAP); (ii) optimize and model the extraction process to maximize extract properties; (iii) chemically characterize the extracts obtained under optimal conditions by GC-MS/FID; and (iv) assess the antifungal activity of the optimized extract against representative white-rot (*Trametes versicolor*) and brown-rot (*Gloeophyllum trabeum*) fungi to evaluate its potential as a bio-based wood preservative.

## Materials and methods

### Materials and reagents

**Fig. 1:**
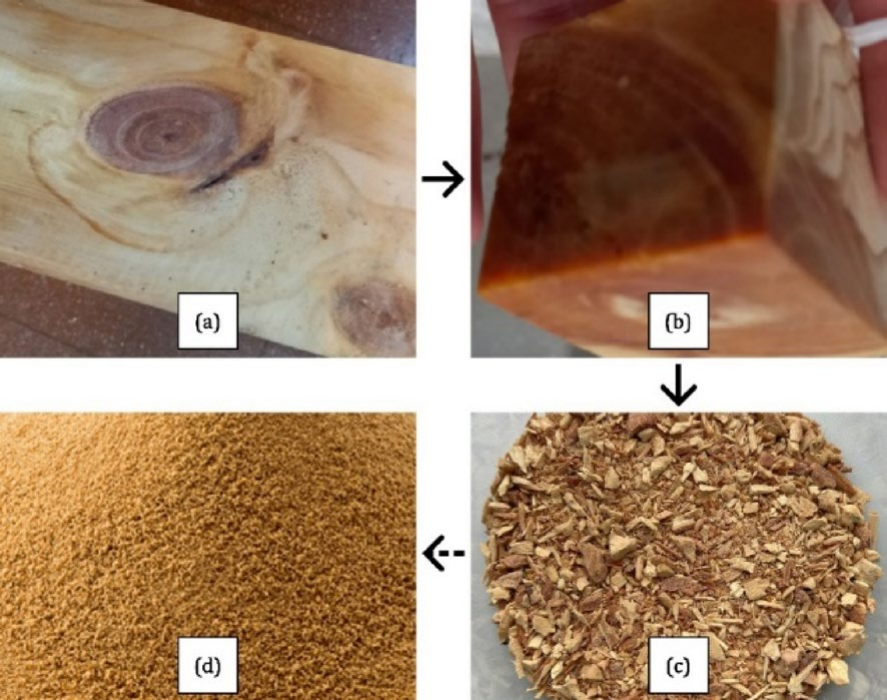
Preparation of knotwood material for characterization and extraction. **a** Board offcuts with knots received from the sawmill. **b** Isolated knots after cutting. **c** Material used for extraction after the first milling. **d** Fraction retained between 20 and 80 mesh sieves after the second milling, used only for raw material characterization

Knotwood samples were supplied by a sawmill located in Rivera, Uruguay, which processes *P. taeda* for the production of clear blanks (finger-jointed), solid finger-jointed boards, and clear boards. They were provided as discarded board offcuts from the trimming of dried sawn wood (shown as (a) in Fig. 1). The offcuts were cut to isolate each knot using an electric saw and subsequently milled in a coarse stage with a 10 mm screen. For chemical characterization, the material was further milled in a cutting mill (IKA MF 10, Germany) equipped with a 2 mm sieve and subsequently sieved; the fraction retained between 20 and 80 mesh was selected. (shown as (d) in Fig. 1), following the National Renewable Energy Laboratory (NREL) procedures (Hames et al. 2008).

**Fig. 2:**
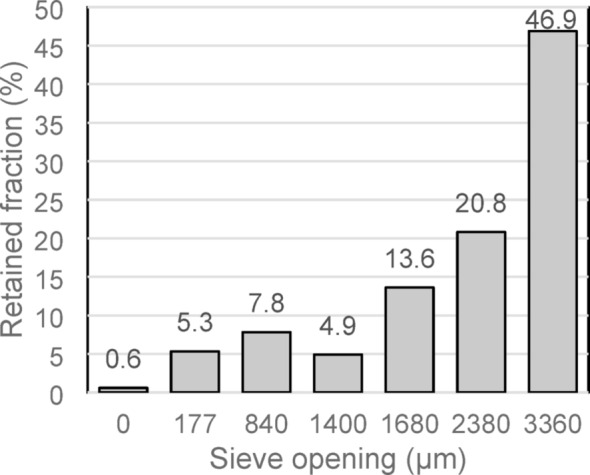
Particle size distribution of the milled knotwood material used for the extractions

For the extractions, the milled material in the first stage was used (showed as (c) in Fig. 1) given the losses of material in the second stage of milling. The particle size distribution of the material used for the extractions is shown in the Fig. 2.

All reagents were of American Chemical Society (ACS) grade or higher. Ethanol, sulphuric acid, hydrochloric acid, sodium carbonate, sodium acetate trihydrate, iron(III) chloride hexahydrate, l-ascorbic acid, gallic acid, Folin–Ciocalteu reagent, agar, malt extract, and the HPLC standards were obtained from Sigma-Aldrich (Steinheim, Germany). 2,4,6-tri(2-pyridyl)-S-triazine (TPTZ) was supplied by Fluka (Steinheim, Germany).

### Compositional analysis

The chemical composition of the raw materials was determined following NREL procedures with some modifications (Sluiter et al. 2005, 2008, 2012). The solid content was determined by drying at 40 °C until constant weight. Total extractives were determined using a Soxtec equipment (Hanon SOX406, China) by subsequent extractions using ethanol, water and acetone, each performed for 90 min (60 min extraction, 30 min rinsing). The extracts were vacuum-dried at 40 °C and the resulting extractive-free solid was acid-hydrolysed for the determination of lignin and carbohydrates. Soluble lignin was quantified in the liquid fraction with a UV spectrophotometer (Shimadzu, Japan) using a wavelength of 240 nm and an absorptive factor of 12 L.g^− 1^.cm^− 1^. Carbohydrates (glucose and the sum of xylose, mannose and galactose -XMG-) were quantified in the liquid fraction, with a HPLC (Shimadzu, Japan) equipped with an Aminex HPX-87 H column (300 × 7.8 mm, Bio-Rad Laboratories Ltd., USA), based on a sulfonated polystyrene–divinylbenzene cation-exchange resin in the H⁺ form., operating at 35 °C with 5 mM H_2_SO_4_ as the mobile phase at a flow rate of 0.6 mL.min^− 1^. The ashes were determined in a muffle furnace at 575 °C. All determinations were performed in duplicate, and the results are expressed as mean ± SD, where SD represents the standard deviation of the replicate measurements.

### Knotwood extractions

The extractions for the preliminary studies and the optimization of the conditions were performed at small-scale in a thermal bath (Biobase, model SWB-A, China), using 100 mL Schott flasks, with orbital agitation (90 rpm), with an extraction time of 90 min for all conditions.

#### Experimental design and optimization study

Prior to the optimization of extraction conditions, a preliminary solvent screening was carried out to support solvent selection. Pure solvents (water, ethanol and acetone) were evaluated under comparable extraction conditions, along with aqueous mixtures (50%, v/v) of ethanol and acetone. In addition, the effect of a hexane pre-extraction step was assessed to determine whether the removal of lipophilic compounds could enhance the extraction of polar constituents. The results showed no significant differences between ethanol and acetone in terms of extraction performance, while the hexane pre-extraction did not improve the recovery of subsequent extracts. Based on these findings, ethanol was selected as the working solvent for further optimization due to its effectiveness, favorable safety and environmental profile, and practical applicability. In particular, ethanol is classified as a GRAS (Generally Recognized As Safe) solvent and is recognized as a safe, non-toxic reagent suitable for the extraction of plant-derived phenolics, while also offering lower operational costs compared to other organic solvents (Venkatesan et al. 2019, 2020). A detailed presentation of this preliminary screening is not included to maintain the focus and conciseness of the manuscript.

Extractions with ethanol–water mixtures were carried out according to a three-factor Box–Behnken design(Box and Behnken 1960) to evaluate the effects of temperature (T), ethanol concentration (EtOH), and liquid-solid ratio (L/S) on EY, TPC, and antioxidant activity FRAP. Each factor was studied at three levels (Table 1), resulting in a total of 12 conditions and three replicates at the central point, with two replicates per experimental run.

**Table 1:** Factors and levels used in the Box–Behnken design for optimization

Code	T (°C) x_1_	EtOH (% (w/w)) x_2_	L/S (w/w) x_3_
− 1	25	50	5
0	50	75	10
+ 1	75	100	15

The experimental data were analysed using second-order polynomial regression models. A backward elimination procedure was applied to retain only statistically significant terms (Montgomery 2017). Statistical analyses were performed with Statistica 14 (TIBCO Software Inc., Palo Alto, CA, USA). For each response ($\:{Y}_{j}$), the selected model had the form:

$$ \begin{aligned} Y_{j} \: = & \:a_{{0j}} \: + \:a_{{1j}} \cdot x_{1} ^{*} \: + \:a_{{2j}} \cdot x_{2} ^{*} + \:a_{{3j}} \cdot x_{3} ^{*} \: + \:a_{{12j}} \cdot x_{1} ^{*} \cdot x_{2} ^{*} \\ & + \:a_{{13j}} \cdot x_{1} ^{*} \cdot x_{3} ^{*} \: + \:a_{{23j}} \cdot x_{2} ^{*} \cdot x_{3} ^{*} \: + \:a_{{11j}} \cdot \left( {x_{1} ^{*} } \right)^{2} \\ & + \:a_{{22j}} \cdot \left( {x_{2} ^{*} } \right)^{2} \:\: + \:a_{{33j}} \cdot \left( {x_{3} ^{*} } \right)^{2} \\ \end{aligned} $$

where $\:{{x}_{1}}^{*}$, $\:{{x}_{2}}^{*}$, $\:{{x}_{3}}^{*}\:$ are the coded independent variables:

$$\:{{x}_{i}}^{*}=\frac{\:({x}_{i}\:-\:{x}_{M})}{\varDelta\:{x}_{i}}$$

with $\:{x}_{i}\:$the actual value of factor i, $\:{x}_{M}$ the value at the design center point, and $\:\varDelta\:{x}_{i}\:$the step (half-range) for factor i. Model adequacy was evaluated by ANOVA at a 95% confidence level (*p* < 0.05). For each dependent variable Yj, experimental values were compared with model predictions and the coefficients of determination (R^2^ and R^2^_adj_) were computed to assess goodness of fit. The optimum of each response was analysed and predicted using the statistical software. Contour plots were generated for each response, and the fitted models were subsequently used to identify the conditions that maximized the responses.

#### Extract properties analysis

After each extraction, the extract was vacuum filtered. To determine the yield of extraction, an aliquot of 10 mL was vacuum-dried at 40 °C until constant weight. The extract was then stored refrigerated (4 °C) for further analysis.

The antioxidant capacity was determined using the FRAP (ferric reducing antioxidant power) method, mixing 0.1 mL of extract with 3 mL of the FRAP working solution. This solution was freshly prepared by combining 25 mL of acetate buffer (300 mM, pH 3.6), 2.5 mL of TPTZ (2,4,6-tri(2-pyridyl)-S-triazine) solution (10 mM in 40 mM HCl), and 2.5 mL of FeCl_3_·6 H_2_O (20 mM). The reaction mixture was incubated for 5 min at 25 °C, after which the absorbance was recorded at 593 nm using a UV spectrophotometer (Shimadzu, Japan). A calibration curve with ascorbic acid was used for quantification, and the antioxidant activity was expressed as nanomoles (nmol) of ascorbic acid equivalents (AAE) per gram of dry sample (Xavier et al. 2025).

The TPC was quantified using the Folin–Ciocalteu assay (Singleton and Rossi 1965). Briefly, 0.5 mL of the extract was mixed with 2.5 mL of Folin–Ciocalteu reagent (previously diluted 1:10, v/v) and 2 mL of sodium carbonate solution (75 g/L). The reaction mixture was kept at 50 °C for 5 min, and the absorbance was measured at 760 nm with a UV spectrophotometer (Shimadzu, Japan). A calibration curve prepared with gallic acid was used, and results were expressed as grams of gallic acid equivalents (GAE) per 100 g of dry material.

#### GC-MS/FID of the optimized extracts

The selected extracts after the extraction optimization (see Sect.  "Extraction for antifungal assays") were analysed by gas chromatography coupled with mass spectrometry and flame ionization detection (GC–MS/FID). The analyses were carried out using an Agilent 6890 GC equipped with an MS 7959 C detector (MSD), FID, and a VF 5-ms column. Separation was performed on a fused silica capillary column (Varian VF-1701ms, 60 m × 0.25 mm × 0.25 μm). The injector was operated in split mode (split ratio 15:1) with an injection volume of 1 µL and injector temperature set at 300 °C. Helium was used as carrier gas at a constant flow of 2.0 mL/min. The oven temperature program started at 45 °C (4 min), followed by a heating rate of 3 °C/min up to 280 °C, where it was held for 20 min, giving a total run time of 102 min. The transfer line temperature was 280 °C. Mass spectra were acquired in scan mode over the range m/z 40–550. FID detection was performed at 280 °C with hydrogen, synthetic air and nitrogen flows of 40, 450 and 45 mL/min, respectively, and a sampling rate of 20 Hz. Identification and quantification of compounds were performed by comparing the spectra with entries from the National Institute of Standards and Techonolgy (NIST) Mass Spectral Library (Version 2.0, 2012) and in-house databases. The identified peaks were transferred to the FID chromatogram, and semi-quantitative analysis was carried out by integrating the chromatographic peaks and determining the resulting relative areas.

#### Extraction for antifungal assays

To obtain sufficient extract for the antifungal tests, two large-scale extractions (K1 and K2) were carried out. The K1 condition was selected to get high extraction yield and total phenolic content and the K2 condition was chosen to produce an extract with high antioxidant capacity (Table 2, see Results and Discussion). Extractions were performed in a 2 L reactor (Parr 4522 M, USA) equipped with a four-blade paddle stirrer operated at 100 rpm to ensure proper mixing. Heating was provided by an electric heating jacket, and the temperature was controlled through a digital controller connected to a water-cooled coil, allowing regulation within ± 1 K. In each extraction, 80 g of dry material were processed.

**Table 2:** Extraction conditions for the antifungal assays

Extract code	T ( °C)	EtOH (% w/w)	L/S (w/w) x_3_	Time (min)
K1	50	100	15	90
K2	75	75	15	90

The extracts were concentrated using a rotary evaporator (Büchi B-480, Switzerland) and subsequently dried under vacuum at 40 °C (Cole Parmer G05053-22, USA).

### Antifungal assays

Antifungal bioassays were carried out based on the poisoned food method with modifications (Balouiri et al. 2016). For this purpose, malt extract agar medium was prepared (malt extract 13 g.L^− 1^, glucose 10 g.L^− 1^, and agar 20 g.L^− 1^). The medium was sterilized and subsequently divided into 300 mL aliquots, to which 0.75 g of extract from each treatment were added to achieve a final concentration of 2.5 g.L^− 1^ in the medium (Alonso 2021). The extracts were previously dissolved in 5 mL of ethanol before incorporation into the medium. A single concentration was selected based on our previous experience as a suitable level for preliminary screening of antifungal activity while minimizing the amount of extract required. As a control, culture medium without knot extracts was used, to which the same amount of ethanol was added. Petri dishes (90 mm) were filled with the prepared media, and each plate was inoculated in the centre with a plug containing actively growing mycelium from pure fungal cultures of *Trametes versicolor* (MVHC 6727) and *Gloeophyllum trabeum* (MVHC 5154). Four replicates were prepared for each fungus and medium. Plates were incubated at 25 °C under alternating light/dark cycles (12 h light / 12 h dark) for 7 days. After incubation, the radial growth of the colony was measured from the edge of the mycelial plug using a calliper. The mean values were calculated, and growth data were plotted. The inhibition of fungal growth (I) was calculated using the following equation (Vek et al. 2020; Vovchuk et al. 2024):

$$ \:I\:\left( \% \right) = \:\frac{{r_{C} - r_{K} }}{{r_{c} }} \times 100 $$

Where *r*_*c*_ is the mean colony radius of the control and *r*_*K*_ is the mean colony radius in the presence of the knotwood extract (measured after 7 days). Inhibition results were expressed as mean ± standard deviation (SD), which reflects the variability among biological replicates. The percentage of inhibition data were analysed by two-way analysis of variance (ANOVA), considering the factors fungal species and extract treatment. Tukey’s HSD test (*p* < 0.05) was applied to identify significant differences among means. Statistical analyses were performed using Statistica 14 (TIBCO Software Inc., Palo Alto, CA, USA).

## Results and discussion

### Knotwood chemical characterization

The knotwood feedstock (93.7 ± 0.5% (w/w) dry basis (d.b.) was characterized in terms of extractives, carbohydrates, lignin, and ash and the results are presented in the Table 3.

**Table 3:** Knotwood characterization results. XMG: sum of xylose, mannose and galactose contents

Component	% (w/w) d.b.
Lignin	23.6 ± 0.4
Acid insoluble lignin	23.2 ± 0.3
Acid soluble lignin	0.4 ± 0.1
Carbohydrates	45.8 ± 2.5
Glucose	28.7 ± 1.7
XMG	16.0 ± 0.6
Arabinose	1.2 ± 0.2
Extractives	30.7 ± 1.2
Ethanol extractives	28.1 ± 1.7
Water extractives	1.8 ± 0.1
Acetone extractives	0.8 ± 0.5
Ash	0.2 ± 0.1

The chemical profile of the material analysed in this work highlights the distinctive composition of knotwood compared to stem wood. For *P. taeda* stem wood from Uruguayan plantations, Palombo et al. (Palombo et al. 2024) reported extractives contents consistently below 5% of dry matter, wit lignin fractions around 25% and carbohydrates xceeding 50%. In contrast, knotood samples exhibited a markedly different composition, characterized by very high extractives (30.7 ± 1.2%(w/w), of which 28. ± 1.7%(w/w) were ethanol-oluble), reduced carbohydrate content (45.8 ± 2.5%(w/w), and comparale lignin levels (23.6 ± 0.4%(w/w). This profil is in agreement with the known chemistry of knotwood, where extractives can reach 40% of dry weight (Kebbi-Benkeder et al. 2015; Nisula 2018; Coniglio et al. 2023b).

### Optimization of extraction conditions

The Box–Behnken design enabled the evaluation of temperature, EtOH concentration, and L/S ratio as independent variables, with EYTPC and antioxidant capacity FRAP as responses. Table 4 shows the conditions of each experiment, as well as the results obtained for the dependent variables, expressed as mean ± SD, where SD represents the standard deviation of the replicate measurements.

**Table 4:** Experimental results of total extraction yield (EY), total phenolic content (TPC) and antioxidant capacity (FRAP), for the experimental design

Exp	T ( °C) x_1_	EtOH (% w/w) x_2_	L/S (w/w) x_3_	EY (% (w/w))	TPC (g GAE/100 g d.b.)	FRAP (mmol AAE/100 g d.b.)
1	25	50	1/10	8.3 ± 0.1	2.1 ± 0.1	8.5 ± 1.1
2	25	75	1/5	15.3 ± 0.6	2.5 ± 0.1	10.6 ± 0.9
3	25	75	1/15	13.2 ± 1.7	2.7 ± 0.6	11.4 ± 0.6
4	25	50	1/10	13.4 ± 0.4	2.4 ± 0.4	9.8 ± 1.6
5	50	50	1/5	11.8 ± 0.1	3.2 ± 0.1	11.0 ± 0.5
6	50	50	1/15	17.5 ± 0.2	5.9 ± 0.4	13.9 ± 0.8
7	50	100	1/5	18.8 ± 0.9	3.7 ± 0.1	9.7 ± 0.1
8	50	100	1/15	19.8 ± 0.7	5.9 ± 0.4	12.9 ± 1.7
9	75	50	1/10	16.9 ± 0.9	4.4 ± 0.4	13.5 ± 1.8
10	75	75	1/5	15.8 ± 1.0	3.2 ± 0.4	9.0 ± 0.7
11	75	75	1/15	17.6 ± 0.3	4.2 ± 0.1	14.7 ± 0.3
12	75	100	1/10	19.6 ± 0.1	4.5 ± 0.2	13.5 ± 0.6
13	50	75	1/10	17.1 ± 0.7	4.2 ± 0.6	13.2 ± 0.1
14	50	75	1/10	18.7 ± 0.4	3.7 ± 0.3	11.7 ± 0.4
15	50	75	1/10	18.9 ± 1.7	3.6 ± 0.6	12.1 ± 0.4

Table 5 shows the regression coefficients of each dependent variable obtained from the coded independent variables. The coefficients of multiple determination ($\:{R}^{2}$) for the regression models describing EY, TPC and FRAP were 0.83, 0.84, and 0.75, respectively, representing a generally acceptable fit of the models, given that models with $\:{R}^{2}\ge\:0.75$ can be considered adequate for response surface optimization (Le Man et al. 2010). Besides, all three models showed *p* values lower than 0.0001, indicating a high level of statistical significance for all response variables.

**Table 5:** Regression coefficients of the second-order polynomial models for each response

Response	Term	Coefficient	*p* value	Significant
Extraction yield (EY)	$\:{\boldsymbol{a}}_{0}$	**18.2**	**< 0.001**	*
	$\:{\boldsymbol{a}}_{1}$	**1.46**	**0.007**	*
	$\:{\boldsymbol{a}}_{2}$	**2.55**	**< 0.001**	*
	$\:{\boldsymbol{a}}_{3}$	0.81	0.060	n.s.
	$\:{\boldsymbol{a}}_{12}$	− 1.46	0.078	n.s.
	$\:{\boldsymbol{a}}_{13}$	0.98	0.107	n.s.
	$\:{\boldsymbol{a}}_{23}$	− 1.16	0.058	n.s.
	$\:{\boldsymbol{a}}_{11}$	**-1.60**	**0.025**	*
	$\:{\boldsymbol{a}}_{22}$	− 0.11	0.870	n.s.
	$\:{\boldsymbol{a}}_{33}$	− 1.16	0.093	n.s.
	Model fit	R^2^ = 0.83; R^2^adj = 0.75; *p* < 0.0001		
Total phenolic content (TPC)	$\:{\boldsymbol{a}}_{0}$	**3.83**	**< 0.001**	*
	$\:{\boldsymbol{a}}_{1}$	**0.67**	**< 0.001**	*
	$\:{\boldsymbol{a}}_{2}$	0.25	0.161	n.s.
	$\:{\boldsymbol{a}}_{3}$	**0.78**	**< 0.001**	*
	$\:{\boldsymbol{a}}_{12}$	− 0.34	0.223	n.s.
	$\:{\boldsymbol{a}}_{13}$	**0.23**	**0.274**	n.s.
	$\:{\boldsymbol{a}}_{23}$	− 0.13	0.539	n.s.
	$\:{\boldsymbol{a}}_{11}$	**− 0.86**	**0.001**	*
	$\:{\boldsymbol{a}}_{22}$	**0.66**	**0.009**	*
	$\:{\boldsymbol{a}}_{33}$	0.15	0.505	n.s.
	Model fit	R^2^ = 0.84; R^2^adj = 0.76; *p* < 0.0001		
Antioxidant capacity (FRAP)	$\:{\boldsymbol{a}}_{0}$	**12.3**	**< 0.001**	*
	$\:{\boldsymbol{a}}_{1}$	**1.09**	**0.006**	*
	$\:{\boldsymbol{a}}_{2}$	0.10	0.788	n.s.
	$\:{\boldsymbol{a}}_{3}$	**1.59**	**< 0.001**	*
	$\:{\boldsymbol{a}}_{12}$	− 0.73	0.222	n.s.
	$\:{\boldsymbol{a}}_{13}$	**1.21**	**0.010**	*
	$\:{\boldsymbol{a}}_{23}$	0.09	0.839	n.s.
	$\:{\boldsymbol{a}}_{11}$	− 0.51	0.304	n.s.
	$\:{\boldsymbol{a}}_{22}$	− 0.09	0.859	n.s.
	$\:{\boldsymbol{a}}_{33}$	− 0.39	0.430	n.s.
	Model fit	R^2^ = 0.75; R^2^adj = 0.64; *p* < 0.0001 = 3.6 × 10–^13^		

ns, non-significant coefficient at a 95%; * *p* value < 0.05. $\:{a}_{0},\dots\:,\:{a}_{33}$ correspond to each coefficient where 1 refers to the variable Temperature, 2 to the variable Ethanol concentration and 3 to the variable Liquid-solid ratio

**Fig. 3:**
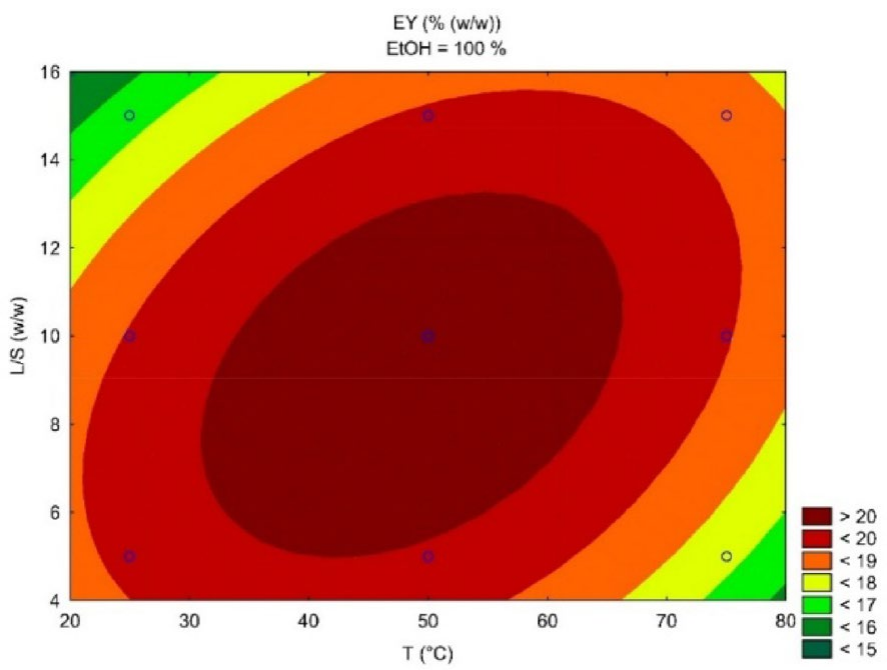
Contour plot showing the effect of temperature (T) and liquid-solid ratio (L/S) on the extraction yield (EY) at a fixed ethanol concentration of 100% (w/w)

EY was mainly influenced by ethanol concentration (x_2_) and temperature (x_1_), both showing significant linear effects (*p* < 0.001 and *p* = 0.007, respectively). The positive coefficients indicate that increasing temperature and ethanol content within the studied range enhanced the extraction efficiency. The quadratic effect of temperature (x_1_^2^, *p* = 0.025) was also significant, revealing the presence of curvature and suggesting the existence of an optimum temperature. Although the L/S ratio and its interactions were not significant, the response surface indicated a slight influence of this factor on extraction yield, suggesting minor combined effects with the other variables. Overall, ethanol concentration exerted the most pronounced effect on EY. Figure 3 shows the effect of temperature and L/S ratio on the EY at a fixed ethanol concentration of 100% (w/w). The EY increased with both L/S ratio and temperature up to an intermediate region, forming an elliptical contour pattern characteristic of a significant quadratic effect with a weaker influence of the L/S. The optimal conditions predicted by the model were a temperature of 47 °C, an ethanol concentration of 100% (w/w), and an L/S ratio of 9 (w/w), under which a maximum EY of 20.8% (w/w) was obtained. Vek et al. (2020) obtained yields between 12 and 17% for *Pinus sylvestris* and *P. niga* knotwood using 70% ethanol–water mixtures at 60 °C while Gérardin et al. (2023) reported similar values (13–17%) for *P. sylvestris* and *Abies alba* knots extracted wit 70–90% ethanol. The higher EY obtained for *P. taeda* knotwod is noteworthy, highlighting its exceptional richness in extractives and confirming its potential as a valuable source of bioactive compounds derived from sawmilling residues.

**Fig. 4:**
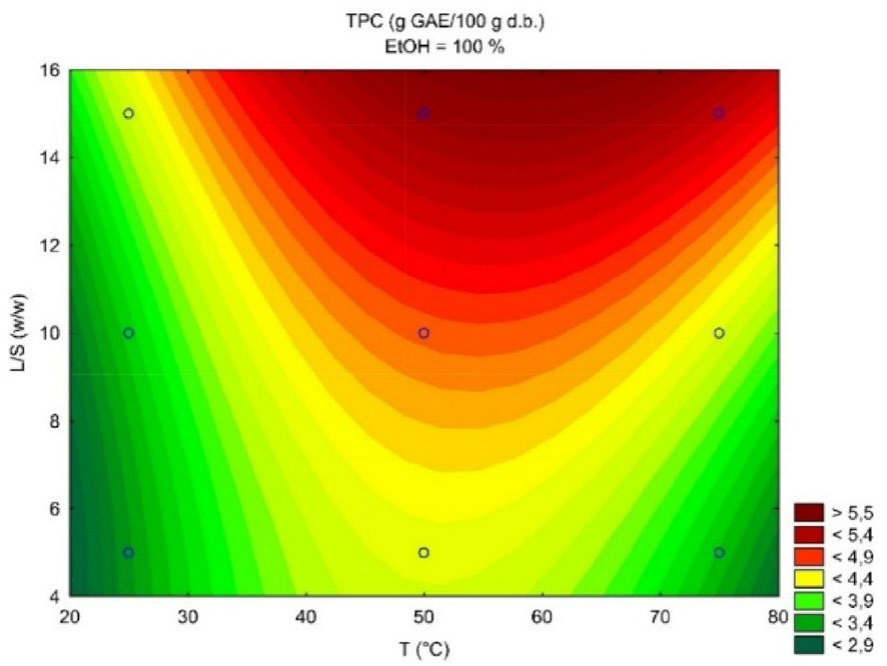
Contour plot showing the effect of temperature (T) and liquid-solid ratio (L/S) on the total phenolic content (TPC) at a fixed ethanol concentration of 100% (w/w)

The TPC was significantly affected by temperature (x_1_, *p* < 0.001), L/S ratio (x_3_, *p* < 0.001), and the quadratic terms of temperature (x_1_^2^, *p* = 0.001) and ethanol concentration (x_2_^2^, *p* = 0.009). The positive linear coefficients for x₁ and x₃ indicate that higher temperature and a larger solvent volume favoured the solubilization of phenolic compounds. The negative coefficient of x₁² points to a decrease at higher temperatures, likely due to partial degradation of thermolabile phenolics, whereas the positive curvature in x₂² suggests a non-linear influence of solvent composition. These results highlight the combined importance of thermal activation and solvent accessibility in maximizing phenolic recovery. The contour plot showing the effect of temperature and L/S ratio on the TPC at a fixed ethanol concentration of 100% (Fig. 4) displays a well-defined curved region, with TPC values increasing up to intermediate-to-high temperatures and larger L/S ratios. The surface shape confirms the significance of the quadratic term for temperature and the positive effect of L/S. The contour map illustrates that phenolic recovery is favoured by high solvent proportion and moderate heating, consistent with the temperature dependence of phenolic compounds solubility and stability. According to the model, the optimal conditions for total phenolic extraction were a temperature of 61 °C, an ethanol concentration of 100% (w/w), and an L/S ratio of 15 (w/w), resulting in a predicted maximum of 5.66 g GAE/100 g d.b. (Table 6). Reported values for other *Pinus* knotwood show a wide range, largely depending on the solvent and analytical approach. Using acetone: water (95:5), Willför et al. (2003b) found that *Pinus contorta* knotwood yielded about 3% total extractives, ith 45% phenolic compounds approximately 1.35 g phenolics/100 g d.b.), while *Pinus sibirica* contained 16% extractives with 79% phenolics (approximtely 12.6 g phenolics/100 g d.b.). In a subsequent study, Willför et al. (2003a) reported that *Pinus sylvestris* knotwood extracted with the same solvent contained 1–7% stilbenes and 0.4–3% lignans, confirmingthe predominance of tese phenolic groups in pine knots. Vek et al. (2020) further quantified around 4 g/100 g d.b. of pinosylvin and pinosylvin monomethyl ether in *Pinus sylvestris* knotwood, meaning that other phenolic compounds might also be present. Although those data were not obtained by the Folin–Ciocalteu method and therefore cannot be directly compared, they indicate a similar magnitude of phenolic enrichment in knot tissues and show that the TPC of *P. taeda* knotwood falls within the upper range reported for pine species.

**Fig. 5:**
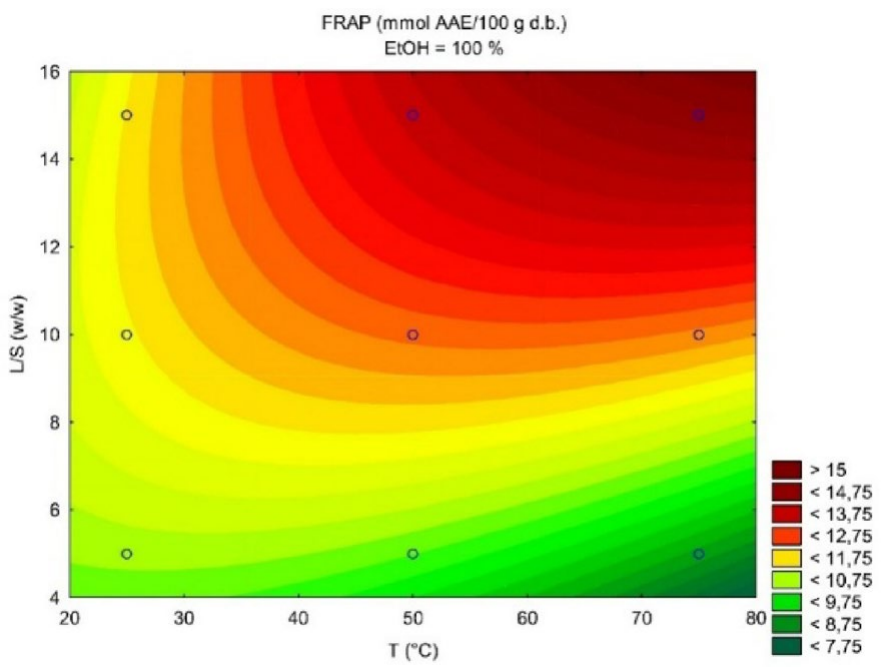
Contour plot showing the effect of temperature and liquid-solid ratio (L/S) on the antioxidant capacity (FRAP) at a fixed ethanol concentration of 100% (w/w)

In contrast, FRAP was primarily governed by temperature (x_1_, *p* = 0.006) and the L/S ratio (x_3_, *p* < 0.001), both exhibiting positive effects. A significant interaction between x₁ and x₃ (*p* = 0.010) indicates that the influence of temperature depended on the extraction scale, suggesting synergistic effects between heat and solvent availability. Quadratic terms were not significant (*p* > 0.05), implying that the responses increased almost linearly within the tested range. EtOH concentration (x_2_) had no significant effect on FRAP (*p* = 0.788), indicating that FRAP was independent of solvent composition within the tested range (50–100% w/w ethanol). This suggests that, once a moderate-to-high ethanol proportion was used, further increases in solvent strength did not enhance the extraction of antioxidant compounds. The contour plot at EtOH of 100% (Fig. 5) shows that FRAP values increased steadily with both temperature and L/S ratio, reaching a maximum at the upper limits of the studied range. According to the model, the optimal conditions for antioxidant activity were a temperature of 75 °C, an ethanol concentration of 100% (w/w), and an L/S ratio of 15 (w/w), yielding a predicted maximum of 15.78 mmol AAE/100 g d.b. (Table 6). Although FRAP is a chemical assay based on ferric ion reduction, similar extractive fractions have also demonstrated potent antioxidant effects in biological systems. Willför et al. (2003b) reported that *Pinus contorta* knotwood extracts exhibited an IC₅₀ of 8.1 µg/mL for inhibiting t-BuOOH-induced lipid peroxidation in rat liver microsomes, almost as effective as Trolox (5 µg/mL) and considerably stronger than BHA (198 µg/mL). The authors attributed this high potency to synergistic interactions among phenolic constituents, as crude extracts outperformed isolated stilbenes and lignans. In addition, Xavier et al. (2021) reported a maximum FRAP value of 24 mmol AAE/100 g d.b. for *P. taeda* bark from Uruguayan plantations under optimized extraction (60 °C, 50% ethanol, L/S = 15). Given that bark extacts have been studied for its high antioxidant activity, the slightly lower but still elevated FRAP obtained for confirms that this sawmilling residue is also a highly potent source of natural antioxidants.

The increase in extractive yield and total phenolic content (TPC) with temperature up to 50 °C can be attributed to enhanced mass transfer, improved solubility of phenolic compounds, and increased diffusivity of the solvent within the matrix. However, at higher temperatures, the observed decrease may be associated with thermal degradation of certain phenolic compounds and/or the extraction of non-phenolic compounds that dilute the overall TPC.

Regarding antioxidant activity, the different trend observed can be explained by the fact that antioxidant capacity does not depend solely on the total concentration of phenolic compounds, but also on their chemical structure and interactions. As reported in the literature, the antioxidant activity of phenolic compounds depends both on individual molecules and on their interactions with other compounds present in the extract (Balasundram et al. 2006). These interactions may be synergistic or antagonistic and can involve other antioxidant species such as reducing sugars, carotenoids, tocopherols, or terpenes (Babbar et al. 2011). Therefore, even if TPC decreases at higher temperatures, the antioxidant activity may remain constant or even increase due to the formation of compounds with higher antioxidant capacity or changes in the interaction between extract components. In this sense, it is important to consider that TPC and antioxidant activity are complementary but not directly correlated parameters, as they are based on different reaction mechanisms (Prior et al. 2005).

**Table 6:** Predicted optimal extraction conditions and responses with 95% prediction intervals for EY, TPC, and FRAP

Response	T (°C)	EtOH (% (w/w)	L/S (w/w)	Predicted value	Prediction interval	Unit
Extraction Yield (EY)	47	100	9	20.8	17.1–24.5	% (w/w)
Total phenolic content (TPC)	61	100	15	5.7	4.4-7.0	g GAE/100 g d.b.
Antioxidant capacity (FRAP)	75	Independent	15	15.8	13.0-18.5	mmol AAE/100 g d.b.

Based on this analysis, two extraction conditions were selected for subsequent GC–MS/FID characterization and antifungal assays (Table 2). Rather than strictly applying the model-predicted optima for each individual response, the selected conditions were defined to represent two contrasting yet practically relevant extraction scenarios, enabling the evaluation of how differences in extract composition influence antifungal activity.

For the first condition (K1), a balanced approach was adopted to obtain a high extraction yield while preserving phenolic compounds. An intermediate temperature of 50 °C, corresponding to the central point of the experimental design, was selected as a practical compromise between the predicted optima for EY (47 °C) and TPC (61 °C), ensuring process robustness while maintaining satisfactory performance for both responses. Since both EY and TPC were maximized at high ethanol concentrations, a solvent concentration of 100% (w/w) ethanol was selected. Regarding the L/S ratio, the highest level (15 w/w) was used, as the response did not vary significantly within the studied range. Although the theoretical maximum predicted by the model was reached around an L/S of 9, it is reasonable to assume that higher solvent volumes favour the extraction of both phenolic and non-phenolic compounds.

For the second condition (K2), the parameters were selected to prioritize high antioxidant capacity (FRAP), choosing the highest temperature (75 °C) and L/S ratio (15 w/w). Although ethanol concentration did not show a significant effect on FRAP, an intermediate value of 75% (w/w) was selected to ensure adequate extraction of phenolic compounds while maintaining a more economically and technically feasible solvent composition.

Overall, the selection of K1 and K2 was intended to generate extracts with distinct chemical profiles, allowing the assessment of how these differences translate into antifungal performance. Table 7 shows the validation of the response variable models. In all cases, the experimental data fell within the model prediction intervals, indicating good predictive ability of the models under the studied extraction conditions.

**Table 7:** Predicted and experimental values of extraction yield, total phenolic content, and antioxidant capacity (FRAP) for the two extraction conditions selected. Predicted values were obtained from the reduced response surface models (± 95% prediction interval), and experimental data are expressed as mean ± SD (*n* = 2)

		Extraction yield (% (w/w))	Total phenolic content (g GAE/100 g d.b.)	Antioxidant capacity (mmol AAE/100 g d.b.)
K1 (50 °C; 100% EtOH; L/S 15)	Predicted	20.8 ± 0.95	5.3 ± 1.3	13.9 ± 2.75
	Experimental	19.4 ± 0.1	3.2 ± 0.4	11.6 ± 1.3
K2 (75 °C; 75% EtOH; L/S 15)	Predicted	18.1 ± 3.3	4.4 ± 1.1	16.2 ± 2.4
	Experimental	21.4 ± 0.1	3.8 ± 0.1	16.0 ± 0.6

In the case of TPC, although the model suggested that, within the confidence interval, a value comparable to the maximum (5.7 g GAE/100 g d.b. at 61 °C) could be obtained, the experimental value was considerably lower. This discrepancy indicates that lowering the temperature from the predicted optimum may have negatively affected the extraction of phenolic compounds. For extract K2, interestingly, both the EY and TPC were higher than those obtained for K1, showing that good phenolic extraction was achieved at both temperatures. Although both conditions resulted in similar EY and TPC, the higher FRAP values obtained at 75 °C and 75% ethanol indicate differences in the composition and antioxidant strength of the extracted compounds.

### GC-MS/FID of the optimized extracts

**Fig. 6:**
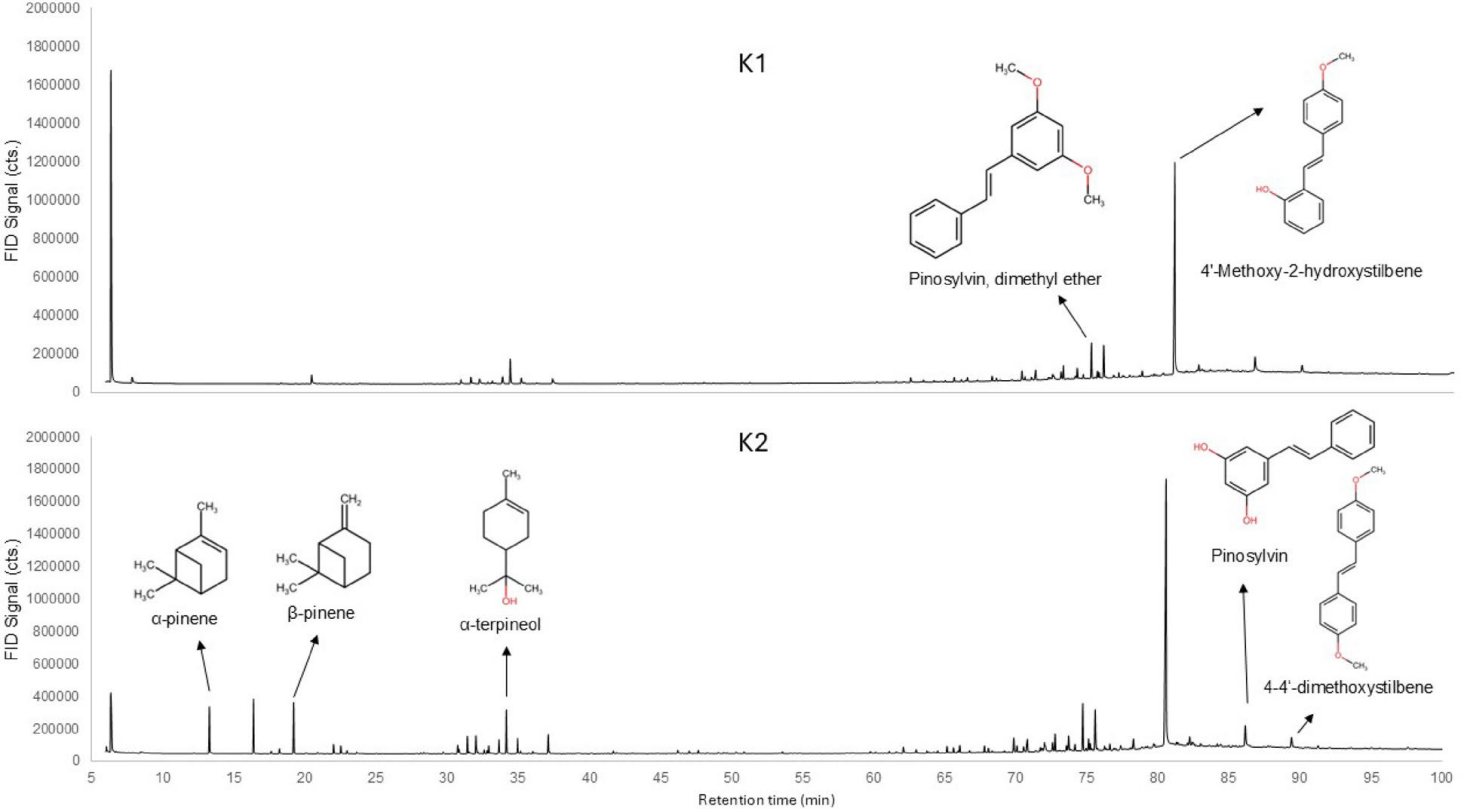
GC-FID chromatograms of the knotwood extracts

The extracts K1 and K2 were analysed by GC–MS/FID to identify compounds potentially responsible for antifungal activity. The GC/FID chromatograms are shown in Fig. 6 and the complete list of compounds detected by MS, together with their relative areas (calculated as the ratio between the peak area of each compound and the total area of all identified peaks in the FID spectra), is presented in Table 8. The relative areas correspond to a semi-quantitative measure and do not represent absolute concentrations.

The results revealed compositional differences between the two extracts. The extract K1 (50 °C, 100% EtOH) showed a markedly high signal for 2-butanone (34.6%) and a much lower relative contribution of terpenes compared with K2. Only six terpenes and twelve terpenoids were detected. At longer retention times, the extract was rich in aromatic compounds, with 4′-methoxy-2-hydroxystilbene (31.1%), pinosylvin dimethyl ether (3.7%), pinosylvin (2.0%), and 4,4′-dimethoxystilbene (1.1%) as the main stilbene derivatives.

In contrast, for extract K2 (75 °C, 75% ethanol), 49 compounds were detected, dominated by 4′-methoxy-2-hydroxystilbene (37.1%), followed by 2-butanone (7.2%) and pinosylvin dimethyl ether (4.3%). A total of 21 terpenes were identified, with α-pinene, β-pinene, and α-terpineol showing the highest relative abundance. Numerous abietane- and pimarane-type terpenoids (e.g., methyl abietate, dehydroabietal, pimarol) were also present, likely reflecting the high resin content typical of pine knots (Nisula 2018). Several aromatic compounds were detected, including two stilbenes—pinosylvin dimethyl ether and 4′-methoxy-2-hydroxystilbene—both known constituents of pine knotwood, with the latter being the most abundant compound in the extract.

The high relative abundance of 2-butanone, particularly in extract K1, was unexpected and its origin remains uncertain. It may be associated with the degradation of wood extractives, such as terpenes or other low-molecular-weight compounds, during the extraction process (Hu et al. 2021). However, this interpretation should be considered with caution, as alternative explanations cannot be excluded, including secondary reactions occurring in the solvent system or potential analytical artefacts related to the injection system or column background. Interestingly, its higher abundance in K1 compared to K2, despite the lower extraction temperature, suggests that factors other than temperature, such as solvent composition or the selective extraction of precursor compounds, may play a role. Further targeted analyses would be required to clarify the formation pathways of this compound.

When considering all aromatic constituents, K2 displayed a higher relative proportion of phenolic compounds, which is consistent with the higher TPC and EY obtained for this extract (Table 7). At higher temperature and 75% ethanol, more phenolic compounds (such as stilbenes) can be extracted. Conversely, K1 contained more low-molecular-weight ketones and fewer terpenes, which is also consistent to the lower yield obtained at the lower temperature compared to K2. In both extracts, stilbenes represented the main phenolic group, accounting for approximately 38% of the total peak area in K1 and 41% in K2.

**Table 8:** GC/MS-FID results for the optimized extracts K1 and K2

RT^a^ [min]	Chemical name	Extract detected	K1 relative area^b^ (%)	K2 relative area^b^ (%)	Group
6.36	Butanone, 2-	K1, K2	34.6	7.2	Ketone
7.84	Butanone, 3-methyl-2-	K1	1.0		Ketone
13.29	Unknown	K2		3.5	Unknown
16.40	α-Pinene	K2	5.1		Terpenes
17.64	Camphene	K2		0.2	Terpenes
18.23	2,4(10)-Thujadiene	K2		0.4	Terpenes
19.24	β-Pinene	K2		4.1	Terpenes
20.37	3-Heptanone, 5-methyl-	K1	1.1		Ketone
22.04	D-Limonene	K2		0.7	Terpenes
22.54	Beta-Phellandrene	K2		0.7	Terpenes
22.97	o-Cymene	K2		0.3	Terpenes
23.66	Benzaldehyde	K2		0.3	Aromatic compounds
30.77	Fenchol	K1, K2	0.5	0.7	Terpenes
31.45	Pinocarveol, trans	K2		1.3	Terpenes
31.46	Endo-Borneol	K1, K2	0.9	1.5	Terpenes
32.04	Cis-Verbenol	K2		1.7	Terpenes
32.06	α-Terpineol	K1, K2	1.9	3.5	Terpenes
32.65	Terpineol	K1, K2	0.2	0.3	Terpenes
32.84	β-Pinone	K2		0.3	Terpenes
32.95	Pinocarvone	K2		0.6	Terpenes
32.96	Unknown	K1	0.3		Unknown
34.21	Unknown	K1	2.8		Unknown
34.99	Phenol, 3-ethyl-	K1	0.8		Aromatic compounds
37.16	Myrtenal	K1, K2	0.6	1.4	Terpenes
35.19	p-Cymen-8-ol	K2		0.2	Terpenes
36.04	Cis-Carveol	K2		0.2	Terpenes
37.14	Verbenone	K2		1.5	Terpenes
47.06	Ethyl 3-phenyl-2-propenoate	K2		0.2	Aromatic compounds
53.63	Unknown	K2		0.2	Unknown
62.13	Hexadecanoic acid, 14-methyl-, methyl ester	K1	0.6		Fatty acids
65.18	Methyl abieta-7,13,15-trien-18-oate	K2		0.7	Terpenoids (abietane type)
65.19	Unknown	K1	0.5		Unknown
65.37	Abietatriene	K2		0.1	Terpenoids (abietane type)
65.66	2-Butenoic acid, (Z)	K2		0.5	Fatty acids
65.67	Unknown	K1	0.2		Unknown
66.09	Methyl 6-dehydrodehydroabietate	K2		0.7	Terpenoids (abietane type)
66.10	Unknown	K1	0.4		Unknown
67.82	Juvabione	K2		0.6	Terpenoids
67.84	1-Cyclohexene-1-carboxylic acid, 4-(1,5-dimethyl-3-oxohexyl)-, methyl ester	K1	0.5		Terpenoids
68.12	Unknown	K2		0.4	Unknown
69.89	2,2’-Binaphthalene	K2		1.4	Aromatic compounds
69.90	Pimaral	K1, K2	1.1	1.7	Terpenoids (pimarane type)
70.11	Unknown	K2		0.5	Unknown
70.57	Unknown	K1, K2	0.5	0.5	Unknown
70.86	Unknown	K1	1.5		Unknown
72.63	Sandaracopimaral	K1	0.9		Terpenoids (pimarane type)
72.80	Isopimara-7,15-dienal	K1, K2	2.1	0.8	Terpenoids (pimarane type)
73.75	8(9),15-Pimarol	K1, K2	1.2	1.4	Terpenoids (pimarane type)
74.20	Dehydroabietal	K1, K2	0.5	0.6	Terpenoids (abietane type)
74.76	Pinosylvin dimethyl ether	K1, K2	3.7	4.3	Stilbenes
75.16	Dehydroabietic acid methyl ester	K1, K2	0.7	0.9	Terpenoids (abietane type)
75.28	Abietal	K1, K2	0.6	0.5	Terpenoids (abietane type)
75.39	Methyl abieta-6,8,11,13-tetraen-18-oate	K2		0.2	Terpenoids (abietane type)
75.61	1-Naphthalenepentanol, decahydro-5-(hydroxymethyl)-5,8a-dimethyl-,2-bis(methylene)-	K2		4.2	Terpenoids
75.61	8(9),15-Isopimarol	K1	4.0		Terpenoids (pimarane type)
76.29	Methyl abietate	K2		0.3	Terpenoids (abietane type)
76.30	Methyl 7,15-Isopimaradien-18-oate	K1	0.4		Terpenoids (pimarane type)
76.65	Unknown	K2		0.5	Unknown
77.42	Dehydro-4-epiabietol	K2		0.7	Terpenoids (abietane type)
78.30	Unknown	K1	0.7		Unknown
78.31	Methyl abieta-8,11,13,15-tetraen-18-oate			1.1	Terpenoids (abietane type)
80.57	4’-Methoxy-2-hydroxystilbene	K1, K2	31.1	37.1	Stilbenes
82.25	15-Hydroxydehydroabietic acid, methyl ester	K1, K2	0.7	0.8	Terpenoids (abietane type)
84.22	7-Oxodehydroabietic acid, methyl ester	K1, K2	0.2	0.2	Terpenoids (abietane type)
86.17	2-(4-Methylphenyl)benzoic acid	K2		2.6	Aromatic compounds
86.18	Pinosylvin	K1	2.0		Stilbenes
89.44	Stilbene, 4,4’-dimethoxy-	K1	1.1		Stilbenes

^a^RT: retention time. ^b^Relative Area: calculated as the relation between the peak area and the total area of the identified peaks. This is a semi-quantitative measure and does not represent absolute concentrations

The composition of extracts K1 and K2 is consistent with the chemical profiles previously described for pine knotwood from several Pinus species. In particular, the predominance of stilbenes such as pinosylvin, pinosylvin dimethyl ether, and 4′-methoxy-2-hydroxystilbene agrees with findings in *Pinus pinaster* knots reported by Gabaston et al. (2017), where these compounds represented the main constituents responsible for the strong antimildew activity against *Plasmopara viticola*. Similarly, Vek et al. (2020) and Jablonsky et al. (2017) identified the same stilbenes, together with minor amounts of lignans and resin acids, in knotwood of *P. sylvestris* and *P. nigra*. In addition to stilbenes, both K1 and K2 contained a wide variety of monoterpenes (α-pinene, β-pinene, limonene, terpineol, borneol) and diterpenoids of the abietane and pimarane series. These compounds have also been reported in pine knot extracts (Willför et al. 2003a; Vek et al. 2020; Gérardin et al. 2023).

Several peaks remained unidentified despite comparison with mass spectral libraries, as no matches with sufficient confidence were obtained. These signals may correspond to more complex or less volatile compounds not adequately resolved under the applied GC–MS conditions. For the major unidentified peaks, tentative classification based on fragmentation patterns suggests that they may correspond to terpenoid- or phenolic-derived compounds; however, only compounds identified with high confidence were considered in the discussion to avoid overinterpretation.

It should be noted that the GC–MS analysis performed in this study was conducted without prior derivatization, which mainly enables the detection of low-polarity compounds. As a result, more polar constituents present in the ethanol extracts, such as sugars, organic acids or polyphenolic compounds, may not have been fully detected or properly characterized. The application of derivatization strategies, such as trimethylsilylation (TMS), would likely improve the detection of these polar compounds. Additionally, complementary analytical techniques such as LC–MS could provide a more comprehensive characterization of the extract composition. These approaches should be considered in future studies to achieve a more complete understanding of the bioactive constituents responsible for the observed antifungal activity.

### Antifungal activity

**Fig. 7:**
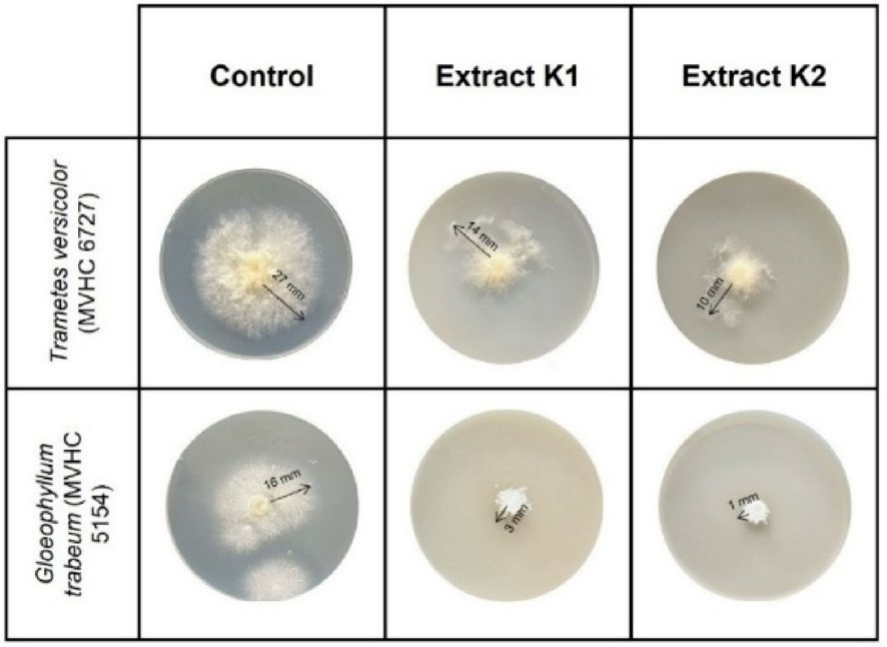
Fungal cultures of Trametes versicolor and Gloeophyllum trabeum after 7 days of incubation in the presence of extracts K1 and K2. Control corresponds to plates without extract. For each condition, a representative plate showing the largest measured colony radius among replicates is presented

**Fig. 8:**
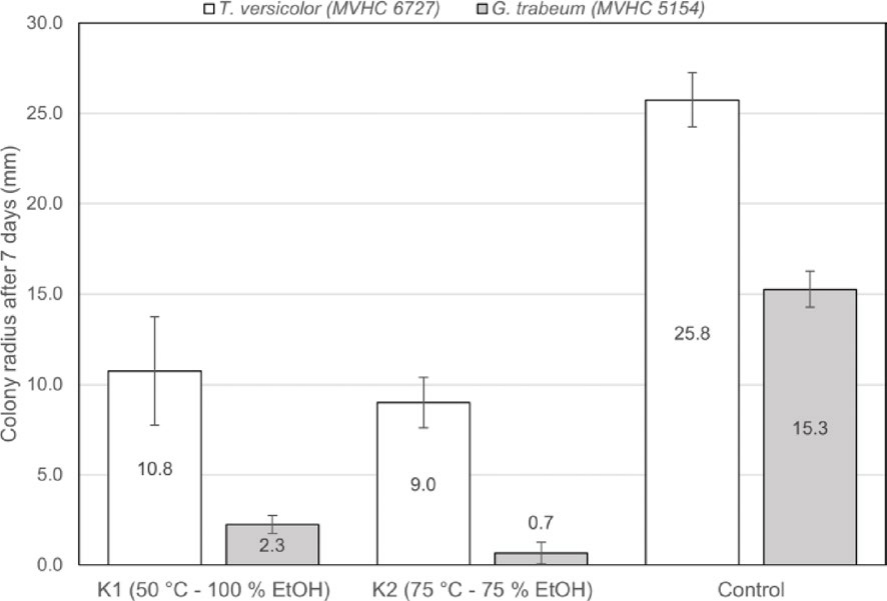
Radial growth of fungal colonies after 7 days of incubation. Values are mean ± SD

After 7 days of incubation, representative plates illustrating fungal growth under each condition are shown in Fig. 7. For each treatment, the plate corresponding to the largest measured colony radius among replicates was selected to provide a clear visual comparison of the antifungal effect of the extracts. The corresponding quantitative results of colony radial growth measured from the mycelial plug are presented in Fig. 8 as mean values with their associated standard deviations. In all cases, the extracts markedly reduced mycelial growth compared to the controls. It should be noted that one plate of *Gloeophyllum trabeum* treated with Extract K2 was discarded due to contamination. Therefore, statistical analyses for this case were performed with *n* = 3 instead of 4.

The percentage of inhibition values are summarized in Table 9. Both extracts were very effective against the brown-rot fungus *Gloeophyllum trabeum*, showing for the extract K2 an almost total inhibition after 7 days. Regarding *Trametes versicolor* the extracts also showed a significant inhibition, reaching more than 60% in for the extract K2 which was more effective than K1 in both cases. According to Tukey’s test, all treatments differed significantly from each other (*p* < 0.05).

**Table 9:** Fungal growth inhibition (%) of knotwood extracts against *Trametes versicolor* (MVHC 6727) and *Gloeophyllum trabeum* (MVHC 5154) after 7 days of incubation

Fungus	Extract	Mean inhibition (%)		
*Trametes versicolor* (MVHC 6727)	K1	58.3^a^	±	11.6
	K2	65.0^b^	±	5.5
*Gloeophyllum trabeum* (MVHC 5154)	K1	85.2^c^	±	3.3
	K2	96.7^d^	±	3.8

Values are mean ± SD, Different letters in the table indicate significant differences according to Tukey’s HSD test(*p* < 0.05)

Although the use of a single concentration provides valuable insight into the antifungal potential of the extracts and is suitable for preliminary screening purposes, it inherently limits the ability to perform a rigorous quantitative comparison of their efficacy. The inclusion of a full dose–response curve, covering a wider range of concentrations, would allow the determination of key parameters such as EC_50_ values and minimum inhibitory concentrations, thereby enabling a more robust assessment of the relative potency of the extracts. In the present study, this approach was not feasible due to the limited availability of extract, which constrained the experimental design. Nevertheless, the selected concentration offers a relevant basis for initial comparison. Future work should therefore focus on evaluating multiple concentrations to establish dose–response relationships and further validate the antifungal performance of these extracts.

The chemical composition of extracts K1 and K2, characterized by high contents of stilbenes, together with diterpenoids and oxygenated monoterpenes, is consistent with the observed antifungal properties. Stilbenes are well-known phytoalexins in conifers, synthesized in response to wounding or microbial infection (Hovelstad et al. 2006; Gabaston et al. 2017). In particular, pinosylvin and pinosylvin monomethyl ether (PME) have been identified as the major active compounds in *Pinus pinaster* knot extracts exhibiting strong antimildew activity against *Plasmopara viticola* (Gabaston et al. 2017). Earlier studies also demonstrated that pinosylvin and PME effectively inhibit the growth of both white-rot and brown-rot fungi such as *Trametes versicolor*,* Phanerochaete chrysosporium*,* Neolentinus lepideus*,* Gloeophyllum trabeum*, and *Postia placenta* (Celimene et al. 1999). These effects are attributed to the capacity of stilbenes to react with sulfhydryl groups of fungal enzymes, impairing metabolic activity (Clausen et al. 1986). In our study, both extracts displayed high antifungal efficacy, particularly against *Gloeophyllum trabeum*, with inhibition values of 85.2% for K1 and 96.7% for K2. While their TPC were comparable (3.2 and 3.8 g GAE/100 g d.b., respectively), extract K2 exhibited a considerably higher FRAP (16.0 vs. 11.6 mmol AAE/100 g d.b.). This observation indicates that the enhanced antifungal performance of K2 is not merely related to a higher phenolic load but rather to the presence of additional non-phenolic redox-active compounds that may potentiate oxidative stress in fungal cells. Recent mechanistic work by Li et al. (2022) confirmed that PME exerts a membrane-disruptive effect by binding to phospholipids, increasing permeability, and causing leakage of intracellular contents. Similar membrane effects were described for pinosylvin by Lee et al. (2017) and further summarized by Bakrim et al. (2022), who reviewed its broad antimicrobial spectrum. In addition, Periferakis et al. (2025) reported that pinosylvin suppresses fungal development by interfering with reactive oxygen species (ROS) homeostasis and mitochondrial activity.

Several studies have confirmed that the antifungal activity of pine knotwood correlates with its stilbene-rich composition. Vek et al. (2020) showed that acetone extracts from *Pinus sylvestris* and *P. nigra* knots, dominated by pinosylvin and PME, inhibited the growth of *Coniophora puteana* and *Trametes versicolor* in vitro. Similarly, Gérardin et al. (2023) observed strong antifungal effects in knot extracts of *Abies alba* and *Picea abies*, where the degree of inhibition was positively related to total stilbene and flavonoid contents. These results are in agreement with the high proportion of stilbenes detected in our K1 and K2 extracts, particularly 4′-methoxy-2-hydroxystilbene, a derivative closely related to PME.

In addition to stilbenes, our extracts contained abundant monoterpenes (for example α-pinene, β-pinene, α-terpineol) and abietane/pimarane diterpenoids. These compounds have also been associated with antimicrobial and fungistatic activity (Hovelstad et al. 2006; Abad et al. 2007). It is reported that several terpenoids, including α-pinene and borneol, inhibit the growth of *Candida spp.* and *Trichophyton spp.*, while resin acids such as dehydroabietic acid can act synergistically with phenolics. The coexistence of these terpenoids with stilbenes in our extracts is consistent with the high resin content typical of pine knots (Hovelstad et al. 2006; Gérardin et al. 2023) and may contribute to the observed antifungal potency.

## Conclusions and future perspectives

This study demonstrated that *P. taeda* knotwood, an abundant by-product of the Uruguayan sawmilling industry, constitutes a rich and underutilized source of bioactive compounds. This material exhibited an exceptionally high extractives content (30.7% w/w, d.b.), dominated by ethanol-soluble components. The extraction process, optimized through a Box–Behnken design, showed that mild conditions using ethanol–water mixtures enabled high extraction yields while maintaining substantial phenolic content and antioxidant activity FRAP. In vitro assays demonstrated strong inhibition of fungal growth, achieving 65% inhibition of *Trametes versicolor* and up to 97% inhibition of *Gloeophyllum trabeum* after seven days. The superior performance of extract K2 correlated with its higher antioxidant capacity, suggesting synergistic contributions from phenolic and non-phenolic compounds.

These results highlight the exceptional richness of *P. taeda* knotwood as a source of bioactive molecules and confirm its antifungal potential comparable to other coniferous species, yet obtained from a low-value industrial residue. From a technological perspective, integrating knotwood valorisation into existing sawmilling processes offers a viable circular approach for producing natural wood protection agents. While the present study provides a reliable preliminary screening of antifungal activity, future work should include dose–response assays to enable a more quantitative comparison between extracts and to determine key parameters such as minimum inhibitory concentrations. In addition, the inclusion of a more comprehensive chemical characterization of the extracts through derivatization-based GC–MS (e.g., TMS) and complementary LC–MS analyses would enable a better identification of polar bioactive compounds and clarify their contribution to antifungal activity. Future work should also address extract impregnation tests, durability evaluation, and process scale-up, including solvent recovery and stabilization of active components, to move toward industrial implementation. Overall, this research demonstrates the potential of *P. taeda* knotwood residues as renewable, sustainable, and locally available raw materials for the development of natural wood protection systems within a bio-based economy framework.

## Data Availability

The datasets used and/or analysed during the current study are available from the corresponding author on reasonable request.
